# Supplementary material for: On the origin of acoustic emission in the stress-induced martensite regime of shape memory alloys
Source: Nat Commun. 2026 Jun 5;17:5015. doi: 10.1038/s41467-026-73946-9 (PMC13241483; doi:10.1038/s41467-026-73946-9)
Supplement: Supplementary file 1 — Supplementary Information [file 41467_2026_73946_MOESM1_ESM.pdf]

Supplementary material for the manuscript

On the origin of acoustic emission in the stress-induced martensite regime of shape memory alloys

C. Lauhoff[a]*, A. Weidner[b], R. Lehnert[b], A. Reul[c], T. Pham[a], M.J. Gutmann[d], P. Krooß[a], W.W. Schmahl[c], H. Biermann[b], H. Seiner[e], T. Niendorf[a]

[a]*Institute of Materials Engineering, Universität Kassel, Mönchebergstr. 3, Kassel 34125, Germany*
[b]*Institute of Materials Engineering, Technische Universität Bergakademie Freiberg, Gustav-Zeuner-Straße 5, 09599 Freiberg, Germany*
[c]*Department of Earth and Environmental Sciences, Applied Crystallography, Ludwig-Maximilians-Universität, Theresienstr. 41, Munich 80333, Germany*
[d]*ISIS Facility, Rutherford Appleton Laboratory, Chilton, Didcot, Oxfordshire OX11 0QX, United Kingdom*
[e]*Institute of Thermomechanics, Czech Academy of Sciences, Dolejškova 5, 182 00 Prague, Czech Republic*

**corresponding author. email: lauhoff@uni-kassel.de; phone: +49 561 804-3976;*

S1 Supplementary figures S-1, S-2, S-3: neutron diffraction patterns

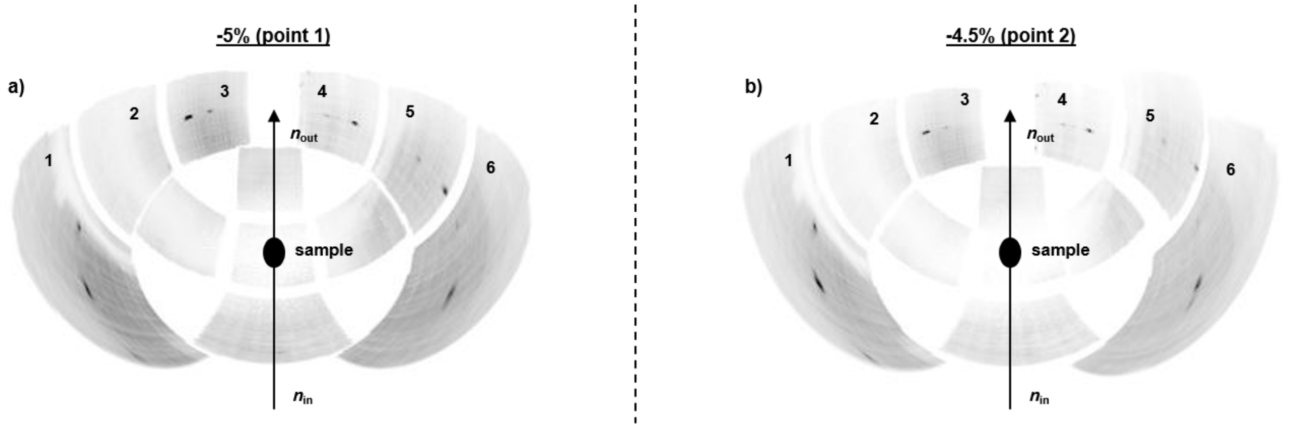

Figure S-1: Complete diffraction patterns collected on the detector arrangement at SXD for single-crystalline ⟨001⟩-oriented Co-Ni-Ga in solution-annealed condition under compression at 100 °C. The diffraction data were recorded during the SE single cycle (loading-unloading) test shown in Fig. 4 in the stress-induced martensitic state at (a) -5% (maximum strain level) and (b) after subsequent unloading to -4.5% applied strain. In the unloaded condition before SE testing, Bragg reflections from the (200) and (002) lattice planes of austenite were centered on high-angle detector 1 and low-angle detector 4 by rotation of the sample around its vertical axis to a fixed position. Furthermore, it should be noted that the detectors positioned beneath the sample location were not accessible during the present experiment due to shading by the miniature load frame. However, these shading effects had no detrimental impact on the determination of the lattice and phase state of the alloy. Reliable analysis could be conducted based on the diffraction data recorded by Laue TOF technique on the remaining six equatorial detectors.

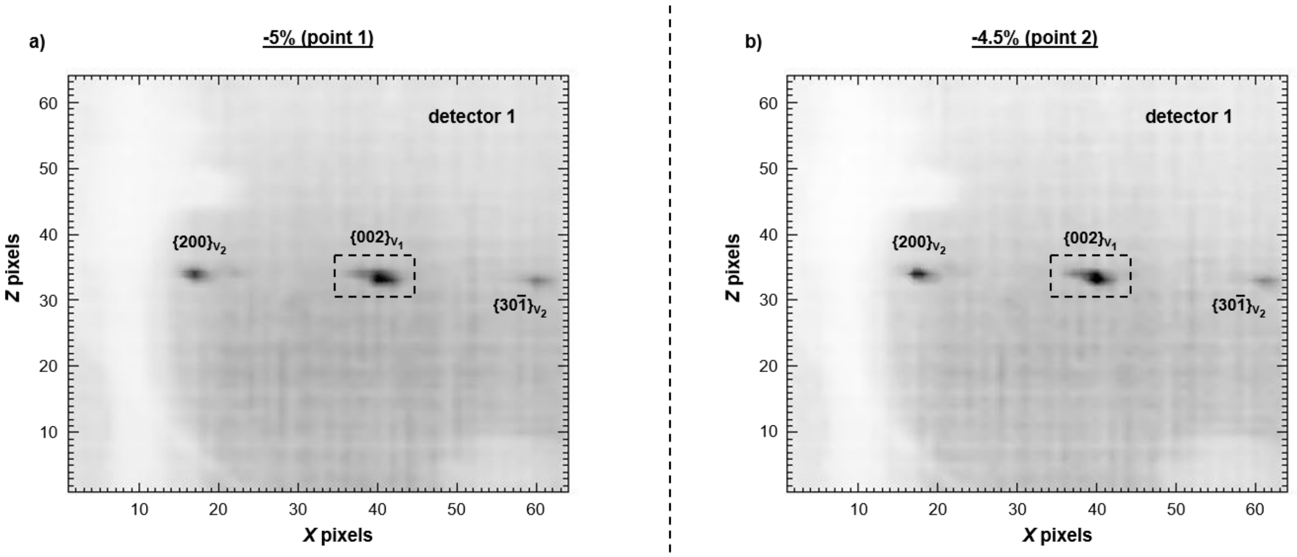

Figure S-2: Diffraction patterns of detector 1 extracted from Fig. S-1: The diffraction data were recorded during the SE single cycle (loading-unloading) test shown in Fig. 4 under compression at 100 °C in the stress-induced martensitic state at (a) -5% (maximum strain level) and (b) after subsequent unloading to -4.5% applied strain. At both strain levels, strong Bragg reflections are visible, indicating a fully martensitic state. All reflections stemming from different lattice planes on detector 1 as well as on the other detectors could be unequivocally attributed to two martensite domain variants, i.e. domain variant V_1 and V_2 forming a twin-related CVP. Intensities related to additional martensite domain variants were not detected.

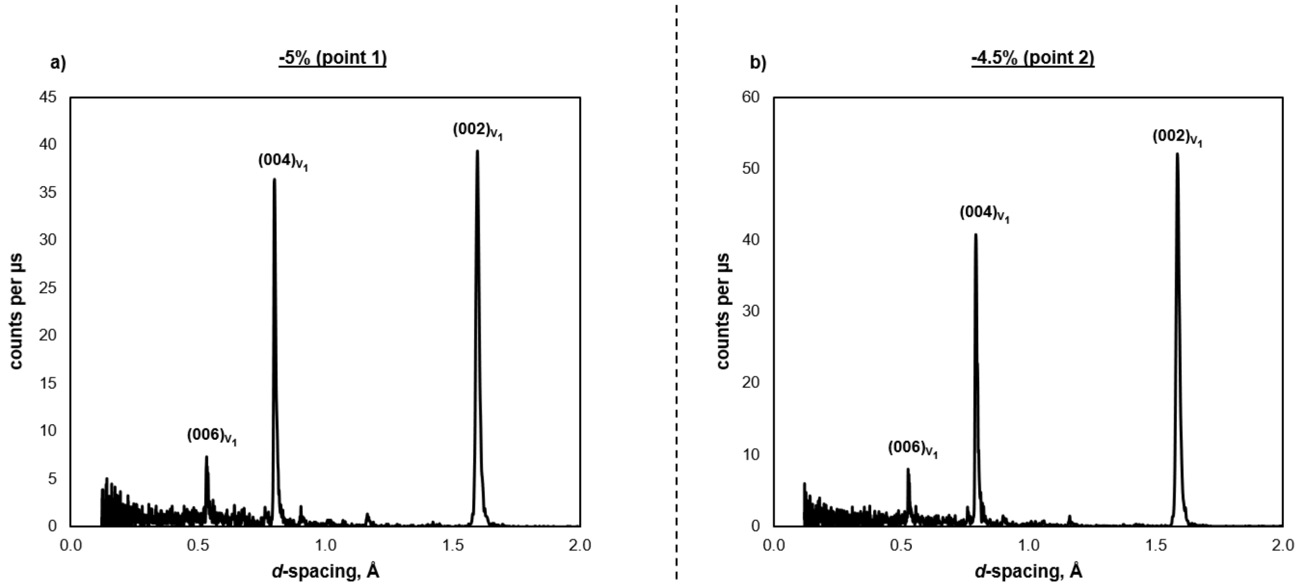

Figure S-3: Indexed 1D-line patterns over $d-$spacing range: The diffraction data were recorded during the SE single cycle (loading-unloading) test shown in Fig. 4 under compression at 100 ˚C in the stress-induced martensitic state at (a) -5% (maximum strain level) and (b) after subsequent unloading to -4.5% applied strain. The 1D-line patterns presented were exemplarily obtained from a single pixel of the martensite Bragg reflections marked correspondingly in Fig. S-2. The 1D-line patterns comprise the intensities of the orders of a given Bragg reflection, e.g. 002, 004, 006. Integration over all detector pixels allows for quantitative phase analysis, i.e. quantification of the domain volume fractions as summarized in Fig. 4b.

S2 Supplementary discussion: compatible martensitic microstructures in Co-Ni-Ga single crystals

In this part of the supplementary information, we summarize the main findings about compatible microstructures that are expected to form in the studied ⟨0 0 1⟩-oriented Co-Ni-Ga single crystals under applied compressive loading. We utilize the formalism of the mathematical theory of martensitic microstructures, as introduced by Ball and James [1, 2] an developed in detail in [3], building upon the phenomenological theory of martensite [4].

S2.1 Notation, theoretical background, and general considerations

We consider a phase transition between cubic austenite with the lattice parameter a_0 and tetragonal martensite with lattice parameters a and c. In the aforementioned theory, the austenite→martensite transition is represented by Bain matrices $\mathbf{U}_I$, with $I = 1, 2, 3$, each for one domain variant of martensite, and of form

$$\mathbf{U}_1 = \begin{pmatrix} e_c & 0 & 0 \\ 0 & e_a & 0 \\ 0 & 0 & e_a \end{pmatrix}, \mathbf{U}_2 = \begin{pmatrix} e_a & 0 & 0 \\ 0 & e_c & 0 \\ 0 & 0 & e_a \end{pmatrix}, \mathbf{U}_3 = \begin{pmatrix} e_a & 0 & 0 \\ 0 & e_a & 0 \\ 0 & 0 & e_c \end{pmatrix}, \tag{1}$$

where $e_a = a/a_0$ and $e_c = c/a_0$.

The theory further assumes that there exists an energy density function $\psi(\mathbf{F})$, being dependent on the deformation gradient $\mathbf{F}$, such that $\psi(\mathbf{F})$ has multiple local minima; these local minima are attained for $\mathbf{F}$ equal (up to a rigid body rotation) to either $\mathbf{U}_I$ or to identity ($\mathbf{I}$), which represents that the material in the given point is in one martensitic domain variant or in austenitic state, respectively. The martensitic microstructure is then expected to be a result of minimization of the integral of $\psi(\mathbf{F})$ over the volume of the whole crystal, subject to boundary conditions.

The energy minimization is typically achieved through formation of *compatible interfaces*, such as twin boundaries (interfaces between different domain variants of martensite) or habit planes (interfaces between austenite and martensite), such that $\psi(\mathbf{F})$ is almost everywhere in one of its local minima.

If two variants of martensite form a twin (also known as a correspondent variant pair, CVP), there exists a rotation matrix $\mathbf{Q}_{IJ}$ such that a so-called rank-one condition is satisfied,

$$\operatorname{rank}(\mathbf{Q}_{IJ}\mathbf{U}_I - \mathbf{U}_J) = 1. \tag{2}$$

This condition mathematically means that the deformation gradients representing the individual variants are identical on one plane, while physically it means that there exists a twinning plane shared by both variants. In the cubic-to-tetragonal transition with Bain matrices (1), the shared planes are always {1 0 1} planes of the tetragonal lattice. The rotation $\mathbf{Q}_{IJ}$ is a small rotation that one variant in the twin needs to undergo to achieve coherency with the second variant at the twinning plane. In tetragonal martensite, the rotation angle is $\phi = 2(\pi/4 - \tan^{-1}(a/c))$.

Similarly, the habit planes are interfaces providing rank-one connections between martensite (typically a mixture of two variants, i.e., internally twinned martensite) and austenite. If $\mathbf{M}$ denotes the effective (mesoscale, homogenized) deformation gradient of martensite, the condition reads

$$\operatorname{rank}(\mathbf{RM} - \mathbf{I}) = 1, \tag{3}$$

requiring a rotation matrix $\mathbf{R}$ to exist. In the most usual case, the martensite at the habit plane is a 1st order regular laminate, which means a microstructure consisting of parallel bands of two variants, connected through twin planes, with mesoscopically constant volume fractions of both variants in the mixture (we will denote the volume fraction of the first variant by κ, where $0 \leq \kappa \leq 1$, while the volume fraction of the second variant is equal to $1-\kappa$). Since the two domain variants in the laminate must satisfy the twinning condition (2), the mesoscopic deformation gradient of the laminate reads

$$\mathbf{M} = \kappa \mathbf{Q}_{IJ} \mathbf{U}_I + (1-\kappa)\mathbf{U}_J. \tag{4}$$

The rank of the matrix in (3) is equal to one, and thus, this matrix can be written as a dyadic product of two vectors $\mathbf{a}$ and $\mathbf{n}$,

$$\mathbf{RM} - \mathbf{I} = \mathbf{a} \otimes \mathbf{n}, \tag{5}$$

that can be chosen such that $|\mathbf{a}| > 0$ and $|\mathbf{n}| = 1$. Then, these two vectors have important physical meanings: $\mathbf{n}$ is a unit vector perpendicular to the orientation of the habit plane, and $\mathbf{a}$ is the so-called *shearing vector* representing the orientation and magnitude of shear strain jump across the habit plane (for transitions with zero or negligible changes in volume, $\mathbf{a}$ is approximately perpendicular to $\mathbf{n}$). The 1st-order laminate (4) that is able to form a compatible habit plane with austenite is sometimes referred to as HPV microstructure, where HPV stands for 'habit plane variants'. Except for alloys satisfying very specific supercompatibility conditions on their lattice parameters [5], the requirement (3) is met only for one duplet $(\kappa, 1-\kappa)$ for each pair of domain variants; and for each volume fraction from this duplet, there exist exactly two solutions [1].

S2.2 Expected microstructures under uniaxial compression

We consider now a crystal in austenite that is loaded in compression along its one principal axis, aligned with the x_3 axis. The microstructure of martensitic domain variants that will form under such loading should be (a close approximation of) an energy minimizer under the following three types of boundary conditions:

1. the total axial contraction of the crystal being set by the grips of the loading device;
2. fixed zero in-plane strains and displacements in the contact area between the crystal and the grips, provided that the crystal cannot freely glide along the grips (which is the case in the reported experiment);
3. compatibility conditions at the internal interfaces, in particular the habit planes, that dictate the volume fractions of the domain variants of martensite close to these interface.

Note that the last condition is not *per se* a boundary condition, but can be understood as one if the energy minimization is considered only for the martensite part of the crystal, as usually done in literature [3]. These three conditions are affecting the microstructure as follows:

Axial contraction of the crystal. Under uniaxial compression along the principal $[0\,0\,1]_{\mathrm{B2}}$ direction of the austenitic parent (B2) phase, only two domain variants of martensite, $I = 1$ and $I = 2$, accommodate the externally imposed contraction along the loading axis, and thus, we can limit our further considerations to these two variants, and denote them as V_1 and V_2, respectively. The only admissible

twinning planes between these two variants are planes being lattice-correspondent to the $(1\,1\,0)_{B2}$ and $(1\,\bar{1}\,0)_{B2}$ planes in austenite. In other words, planes that are parallel to the loading (x_3) direction and inclined by approximately $\pi/4$ from the lateral surfaces of the sample. The ratio between the volume fractions of V_1 and V_2 does not affect the axial contraction of the sample, which means the first boundary condition has no effect on this ratio. The axial compression also adds small elastic compressive strains to the lattice, resulting in perturbation of e_c and e_a that may affect the strain compatibility. However, as the [0 0 1]-oriented Young's moduli of both phases are typically not below 10 GPa as known from similar alloys, and the transformation stress in the experiments reported in the main text does not significantly exceed 200 MPa, the elastic strains are 2 % at maximum (which means one order of magnitude smaller than the transformation strains), having practically no impact on the compatibility between the phases, and we will neglect this effect in further analysis.

Contact areas between the crystal and the grips. The consequences of the rigid contact between the sample and the grips are two-fold. It prevents (i) the in-plane extensions or contractions in the cross-sections close to the grips, and (ii) rigid-body motions of these cross-sections along the grips. The latter condition affects the progress of the stress-induced phase transition, as discussed below. The former condition, in turn, remains identically satisfied (up to some small elastic strains) throughout the transition as long as the material in the contact area remains in austenite, and thus, affects mainly the terminal stages of the experiment. When the sample fully transforms to a mixture of domain variants V_1 and V_2, the resulting in-plane strains can be expressed (in a small strains/small-rotations approximation) as

$$\begin{aligned} \varepsilon_{11} &= \kappa(e_c - 1) + (1-\kappa)(e_a - 1), \\ \varepsilon_{22} &= \kappa(e_a - 1) + (1-\kappa)(e_c - 1), \end{aligned} \tag{6}$$

where κ is the volume fraction of variant V_1 in the mixture. Summing the expressions (6) results in an affine combination condition for ε_{11} and ε_{22} of

$$\varepsilon_{11} + \varepsilon_{22} = e_a + e_c - 2 = \text{const.}. \tag{7}$$

This behavior is schematically visualized in Fig.S-4a, showing that the cross-section elongates in the x_1 direction if V_1 is dominant in the mixture, and in the x_2 direction if V_2 is dominant in the mixture, while the cross-section expands isotropically for $\kappa = 1/2$ (1:1 mixture). To satisfy the boundary condition, we require $\varepsilon_{11} = \varepsilon_{22} = 0$, which means that the spontaneous strains given by the equations (6) above need to be compensated by elastic straining of the material in the cross-section. Assuming that the mixture of variants V_1 and V_2 is isotropic in the x_1x_2 plane in terms of elastic moduli, it can be easily shown that a minimum of the strain energy in the cross-section is attained for $\kappa = 1/2$, because the strain energy is a quadratic function in the strains. That is, the boundary condition at the grips forces the martensite to be composed of equal volume fractions of both domain variants. Further reduction of the strain energy is possible if the cross-sections adjacent to the grips are not fully transformed, i.e., if there is some residual austenite, or if the third variant appears in the microstructure close to the grips. This means that at the grips the boundary condition from the contact area competes with the driving force from the above axial boundary condition that favours all austenite to be transformed to variants V_1 and V_2. For symmetry reasons, nevertheless, even if there is residual austenite close to the grips, or if the third variant appears there, the energy minimum is always attained for equal volume fractions of V_1 and V_2.

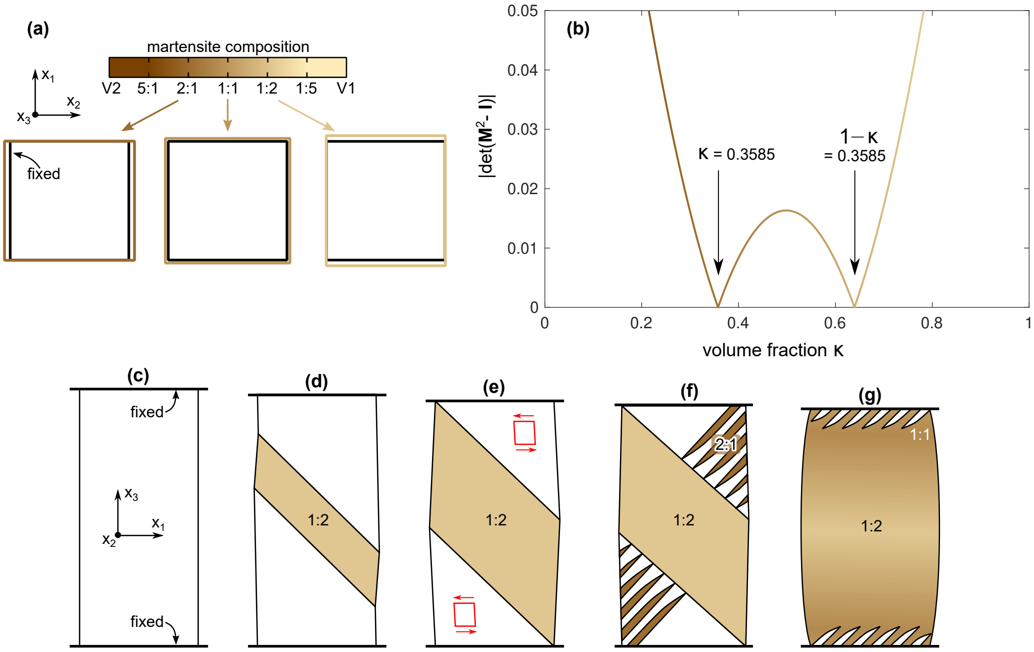

Figure S-4: (a) The effect of the ratio V_2:V_1 (in terms of volumes) on the cross-section of the crystal and its difference from the cross-section of initial austenite, that is fixed in the contact area between the crystal and the grips; the smallest misfit in terms of energy is for 1:1. (b) The scalar incompatibility measure $\det(\mathbf{M}^2-\mathbf{I})$ as a function of the volume fraction κ; the minima are attained for approximate V_2:V_1 ratios of 1:2 and 2:1. (c-g) Tentative evolution of the microstructure in an axially loaded crystal under competing boundary conditions from (a) and (b); a band with 1:2 mixture of martensite domain variants spreads over the whole crystal, inducing secondary shear stresses in the sample (red arrows in (e)). At the stage (f), the band starts interacting with the fixed ends of the sample, which may lead to more complex microstructures appearing close to the grips, eventually variations of the V_2:V_1 ratio in it (g). The color scalebar visualizing the V_2:V_1 ratio introduced in (a) is then used throughout the whole figure.

Conditions imposed by the habit planes. Unlike the previous two conditions, the condition of compatibility at the habit planes is dependent on the exact lattice parameters. The lattice parameters of austenite and tetragonal martensite for the studied alloy are a_0=2.879 Å, a =2.737 Å and c =3.157 Å, which gives the Bain distortion ratio of $c/a = 1.154$ and the volume change $\delta V < 0.9$ %, both values comparable to those of other Heusler-type shape memory alloys.

The Bain matrix components calculated from the lattice parameters are $e_a = a/a_0 = 0.9507$ and $e_c = c/a_0 = 1.0966$. For a coordinate system set such that the x_3 axis is parallel to the loading direction, i.e., the [001] direction in the parent phase, the twinning planes between the variants V_1 and V_2 are approximately parallel to planes $x_1 = x_2$ and $x_1 = -x_2$, which corresponds to the twinning planes $(1\,0\,1)$ and $(1\,0\,\bar{1})$ in the tetragonal lattice (all these planes are lattice-correspondent to $(1\,1\,0)_{B2}$ or $(1\,\bar{1}\,0)_{B2}$ planes of the parent austenitic phase). These two twinning planes are crystallographically equivalent with respect to austenite, which means that the laminates built using these twinning planes will satisfy the habit plane rank-one condition (3) for the same (κ, $1-\kappa$) duplet. To find this duplet, the equation (3) cannot be directly used, as it is a discrete algebraic equation unsuitable for numerical treatment. For this reason, several continuous scalar measures have been introduced in the literature that represent the magnitude of deviation of the rank of the matrix $(\mathbf{RM} - \mathbf{I})$ from one. The most detailed is the so-called '$\lambda_2 = 1$' measure [8] that uses eigenvalues of the difference between matrices $\mathbf{M}$ and $\mathbf{I}$. For our purpose, however, a simpler measure introduced by Forclaz [9] as $\det(\mathbf{M}^2 - \mathbf{I})$ is sufficient; the compatibility can be achieved if and only if $\det(\mathbf{M}^2 - \mathbf{I}) = 0$. The result of the calculation is shown in Fig.S-4(b), where it is seen that

the compatibility is achieved for $\kappa = 0.3585$ (and for the corresponding $1-\kappa$). Notice that $\kappa \approx 1/3$ is a typical value for HPV laminates for shape memory alloys with small volume change between austenite and martensite, and it represents the ratio between volumes of V_2 and V_1 equal to 1:2 (the second solution is 2:1 for $\kappa = 1 - 0.3585 = 0.6415$).

The found value of κ allows us to find the explicit orientations of the habit planes for all possible eight HPV laminate families that can satisfy the compatibility condition, and the corresponding orientations and magnitudes of the shearing vectors. These results are summarized in Table S-1. It is seen that all habit plane orientations are crystallographically equivalent, i.e., from the first $\mathbf{n}$ and $\mathbf{a}$, the other seven sets can be obtained by applying the symmetry elements of the cubic lattice.

From this explicit calculation, the main observation is that all habit planes between austenite and mixtures of domain variants V_1 and V_2 run along approximately $(0\,{\pm}1\,{\pm}1)$ planes, and that they dictate the volume fraction of V_1 in the stress-induced martensite to be $\kappa \approx 1/3$ or $\kappa \approx 2/3$, which competes with the energy-minimization condition $\kappa \approx 1/2$ following from the contact area between the sample and the grips at terminal stages of the loading run.

S2.3 Proposed martensitic phase transition mechanism under given boundary condition

By combining the inputs from the experiments (see main text) and the results of the model calculations summarized above, we can propose a progress of the stress-induced phase transition is as outlined in Fig.S-4(c-g). This progress is idealized, assuming that all boundary conditions are exactly satisfied and neglecting the effect of small elastic strains due to the axial force. Nevertheless, it enables us to interpret the main features observed in the experiment.

As a starting configuration, we assume a crystal that is fixed in the contact areas between the sample and the loading device and loaded axially in compression along the x_3 axis, Fig.S-4c. With increasing axial contraction, a band of martensite (HPV plate) is formed (Fig.S-4d); the band is connected to austenite through habit planes, and the volume fraction of variant V_1 in the stress-induced martensite is given by the compatibility condition, so that the ratio V_2:V_1 is approximately either 1:2 or 2:1. We choose 1:2 for the sketch, for reasons rationalized below. The macroscopic strain carried by the band is pure shear strain given by the shearing vector $\mathbf{a}$ pointing parallel to the habit plan, and the crystal becomes slightly 'crooked'. The martensite can nucleate as a single band, or as multiple parallel bands. In the optical micrographs in the main text (Fig. 2), it is seen that few bands appear during the forward transition,

Table S-1: All possible habit plane orientations $\mathbf{n}$ and shearing vectors $\mathbf{a}$ for 1st-order laminates composed of variants V_1 and V_2. For each orientation of the twinning planes ($(1\,1\,0)_{B2}$ vs. $(1\,\bar{1}\,0)_{B2}$ in terms of lattice-correspondent planes in the parent austenitic phase) and each volume fraction of V_1 in the laminate (0.3585 vs. 0.6415), there are exactly two solutions.

No.	Twinning plane	κ	$\mathbf{n}$	$\mathbf{a}$
1	$(1\,1\,0)_{B2}$	0.3585	[-0.0778 0.6842 -0.7252]	[-0.0068 0.0597 0.0694]
2	$(1\,1\,0)_{B2}$	0.3585	[0.0778 -0.6842 -0.7252]	[0.0068 -0.0597 0.0694]
3	$(1\,\bar{1}\,0)_{B2}$	0.3585	[0.0778 0.6842 -0.7252]	[0.0068 0.0597 0.0694]
4	$(1\,\bar{1}\,0)_{B2}$	0.3585	[0.0778 0.6842 0.7252]	[0.0068 0.0597 -0.0694]
5	$(1\,1\,0)_{B2}$	0.6415	[-0.6842 0.0778 -0.7252]	[-0.0597 0.0068 0.0694]
6	$(1\,1\,0)_{B2}$	0.6415	[0.6842 -0.0778 -0.7252]	[0.0597 -0.0068 0.0694]
7	$(1\,\bar{1}\,0)_{B2}$	0.6415	[0.6842 0.0778 0.7252]	[0.0597 0.0068 -0.0694]
8	$(1\,\bar{1}\,0)_{B2}$	0.6415	[0.6842 0.0778 -0.7252]	[0.0597 0.0068 0.0694]

but not numerous fine bands as observed during the reverse one. For this reason, we use a single band for a schematic visualization of the process at this point. We also assume that the band nucleates in the middle of the sample, because of the boundary conditions in the contact areas that are supposed to suppress the nucleation in the area of the grips. This holds true when the contacts are fully fixed, which is an idealized boundary condition. In real experiments, instead, the edges of the contact areas may act as stress concentrations and the bands may nucleate from there.

With growing the martensite band, the change of the shape of the sample becomes more pronounced (the 'crookedness' becomes stronger), which competes with the zero-displacement boundary condition in the contact areas. As a result of the fixed contact areas, shear elastic stresses arise in the sample (Fig. S-4e), compensating the lateral shape change. These shear stresses can initiate nucleation of alternately oriented martensite (HPV) bands with opposite orientations of the shearing vectors and reversed V_2:V_1 ratio, which would lead to formation of complex microstructures, as observed for example in our previous study [10]. Here, however, the optical microscopy observations do not confirm such a scenario – it is plausible that minor HPV bands with a second orientation appear only in the very final stages of the band growth, for example when the edges of the band reach the grips (Fig. S-4f), and in minor volumes that were not resolvable by the neutron diffraction measurements. The minor martensite bands as sketched in (Fig. S-4f) relax the shear strains and contribute to the overall axial contraction of the sample, however, without violating the zero-strain condition in the contact areas.

To accomplish the stress-induced phase transition, finally, the major HPV band needs to merge with the minor bands in order to proceed towards the contact areas between the sample and the grips, while complying at the same time with the zero in-plane strain boundary condition applying there. The merging can be expected to affect the V_2:V_1 ratio throughout the sample, as the regions with ratios 1:2 ($\kappa = 0.6415$, the major band) and 2:1 ($\kappa = 0.3585$, minor bands) coalesce, and locally they need to reach the ratio of 1:1 ($\kappa = 0.5$) at the grips, where it is also highly plausible that some residual austenite persists. The result is schematically sketched in Fig.S-4g, where it is assumed that the V_2:V_1 ratio throughout the crystal varies between 1:2, inherited in the central parts of the sample from the major band, and 1:1 enforced by the boundary condition at the contact areas.

The experimentally observed ratio of V_2:$V_1 \approx 0.7$ ($\kappa = 0.5882$) supports the proposed scenario, especially if we assume that the major HPV band has the 1:2 (not 2:1) ratio between the volumes of the variants. The exact mechanism how the major band grows further towards the grips, possibly merges with the minor bands (if these indeed arise in terminal stages of the experiment), and finally accommodates the contact area boundary conditions is not known and can be extracted neither from this simplified model, not from the experimental data. However, the neutron diffraction results for the fully compressed sample (-5% compressive strain) clearly reveal only reflections stemming from two dominant martensitic domain variants (Figs. S-2 and S-3) with a fixed rotation between them (that is, with one rotation matrix $\mathbf{Q}_{IJ}$), which means the crystal is dominantly composed of a single 1st-order laminate. This probably means that the major band indeed spreads over the whole crystal, and, when approaching the grips, it gradually changes the V_2:V_1 ratio inside to accommodate the contact-area boundary conditions. As these conditions are not ideal (e.g., some gliding is possible, but at the expense of energy dissipation by friction at the contacts) and because the rearrangement of the domain variants inside the laminate is hysteretic, the 1:1 ratio is fully reached in the terminal state, but the resulting average κ leans towards 0.5. It can also be true that the crystal is either not ideally cut, or not ideally loaded: any deviation from the exact axial $[0\,0\,1]-$orientation of the loading may prefer one orientation of the habit plane over other, and could also affect the optimal terminal V_2:V_1 volume ratio.

In the terminal stage (Fig.S-4g), the sample is elastically stressed, possibly exhibiting 'barelling', i.e., showing larger cross-sectional expansion in the central part than close the grips where the boundary conditions hinder the lateral displacements and strains. Given this scenario, the microstructure inside the crystal can be expected to evolve further with any, even quite small changes of the boundary conditions. Under unloading from -5% to -4.5% compressive strain, the V_2:V_1 ratio further evolves. For example, the existence of small volume fractions of residual austenite in areas close to the grips may locally introduce habit planes enforcing the 2:1 ratio in their vicinity, which turns to further lowering of the effective κ. On the unloading plateau, in turn, the martensitic microstructure in the crystal is already quite far from the first order laminate with the 1:2 ratio created by the initial major HPV band during the forward transition, and thus, the reverse transition proceeds differently. The bands with slightly different orientations and different shadowing contrasts seen in Fig. 2f of the main document suggest that several types of habit planes from Table S-1 may appear, most probably, again, to avoid extensive gliding of the sample in the grips.

Final remarks

As mentioned above, the used boundary conditions are idealized, and, at the same time, the way how they are applied to the system is significantly simplified. Most importantly, their effect is assumed to be sequential, i.e., applied one after another: Firstly, the axial motion of the grips initiates stress-induced transition of parent austenite into a V_1+V_2 mixture of martensite domain variants by forming an HPV band in the middle of the sample; secondly, the habit plane rank-one condition determines the V_2:V_1 ratio inside of the created band to be approximately 1:2; finally, the growing band with the fixed V_2:V_1 ratio starts interacting with the fixed-end conditions at the grips, first by inducing elastic shear stresses, and later by accommodating the zero-strain condition in the contact area between the sample and the grips by changing the V_2:V_1 ratio. In this sense, the proposed process phase transition process bridges between three subsequent energy minimizers: (i) pure austenite, (ii) twinned martensite with a 1:2 domain volume ratio forming a band encapsulated by two habit planes, and (iii) twinned martensite with a 1:1 domain volume ratio that minimizes energy with respect to the fixed-end boundary conditions.

In the real experiment, in contrast, there exists an energy-minimizing path that continuously connects there three stages. Right after the stress-induced nucleation, the twinned martensite inside of the major HPV band is immediately affected by the fixed-end boundary condition, and thus, the V_2:V_1 is assumed to change towards 1:1 as the band propagates towards the ends of the sample. Moreover, in case the experiment would be cyclically repeated, local defects and stress concentrations facilitating the transition may appear in the crystal. This 'training effect' further affects the evolution of the microstructure, and may lead to a deviation of the real process from the theoretical energy-minimizing pathway presented.

References

[1] Ball, J.M., James, R.D. Fine phase mixtures as minimizers of energy (1987) Archive for Rational Mechanics and Analysis, 100 (1), pp. 13 - 52 DOI: 10.1007/BF00281246

[2] Ball, J.M., James, R.D. Local minimizers and phase transformations (1996) ZAMM Zeitschrift fur Angewandte Mathematik und Mechanik, 76 (SUPPL. 2), pp. 389 - 392

[3] Bhattacharya, K, Microstructure of Martensite (2003) New York: Oxford University Press.

[4] Wayman, C.M. The phenomenological theory of martensite crystallography: Interrelationships (1994) Metall and Mat Trans A 25, 1787 - 1795

[5] Gu, H., Bumke, L., Chluba, C., Quandt, E., James, R.D. Phase engineering and supercompatibility of shape memory alloys (2018) Materials Today, 21 (3), pp. 265 - 277 DOI: 10.1016/j.mattod.2017.10.002

[6] Bhattacharya, K. Wedge-like microstructure in martensites (1991) Acta Metallurgica et Materialia, 39 (10), pp. 2431 - 2444 DOI: 10.1016/0956-7151(91)90023-T

[7] Hane K. F. Microstructures in thermoelastic martensites (1998). PhD thesis University of Minnesota

[8] Cui, J., Chu, Y.S., Famodu, O.O., Furuya, Y., Hattrick-Simpers, J., James, R.D., Ludwig, A., Thienhaus, S., Wüttig, M., Zhang, Z., Takeuchi, I. Combinatorial search of thermoelastic shape-memory alloys with extremely small hysteresis width (2006) Nature Materials, 5 (4), pp. 286 - 290 DOI: 10.1038/nmat1593

[9] Forclaz, A. Simple criterion for the existence of rank-one connections between martensitic wells (1999) Journal of Elasticity, 57 (3), pp. 281 - 305 DOI: 10.1023/A:1007697701944

[10] Lauhoff, C., Reul, A., Langenkämper, D., Krooss, P., Somsen, C., Gutmann, M.J., Pedersen, B., Kireeva, I.V., Chumlyakov, Y.I., Eggeler, G., Schmahl, W.W., Niendorf, T. Effects of aging on the stress-induced martensitic transformation and cyclic superelastic properties in Co-Ni-Ga shape memory alloy single crystals under compression (2022) Acta Materialia, 226, art. no. 117623, DOI: 10.1016/j.actamat.2022.117623
